# Passive sensing on mobile devices to improve mental health services with adolescent and young mothers in low-resource settings: the role of families in feasibility and acceptability

**DOI:** 10.1186/s12911-021-01473-2

**Published:** 2021-04-07

**Authors:** Sujen Man Maharjan, Anubhuti Poudyal, Alastair van Heerden, Prabin Byanjankar, Ada Thapa, Celia Islam, Brandon A. Kohrt, Ashley Hagaman

**Affiliations:** 1 Transcultural Psychosocial Organization (TPO) Nepal, Kathmandu, 44600 Nepal; 2 Division of Global Mental Health, Department of Psychiatry and Behavioral Sciences, George Washington School of Medicine and Health Sciences, 2120 L St NW Suite 600, Washington, DC 20037 USA; 3 Center for Community Based Research, Human Sciences Research Council, Pietermaritzburg, South Africa; 4 Medical Research Council/Wits Developmental Pathways for Health Research Unit, Department of Pediatrics, Faculty of Health Sciences, University of the Witwatersrand, Johannesburg, South Africa; 5 George Washington School of Medicine and Health Sciences, Washington, DC 20037 USA; 6 Department of Social and Behavioral Sciences, Yale School of Public Health, Yale University, New Haven, CT 06510 USA; 7 Center for Methods in Implementation and Prevention Science, Yale School of Public Health, Yale University, New Haven, CT 06510 USA

**Keywords:** Adolescent, Child health, Depression, Developing countries, Digital health, Digital phenotype, Mental health, Postpartum depression, Psychotherapy, Nepal

## Abstract

**Background:**

Passive sensor data from mobile devices can shed light on daily activities, social behavior, and maternal-child interactions to improve maternal and child health services including mental healthcare. We assessed feasibility and acceptability of the Sensing Technologies for Maternal Depression Treatment in Low Resource Settings (StandStrong) platform. The StandStrong passive data collection platform was piloted with adolescent and young mothers, including mothers experiencing postpartum depression, in Nepal.

**Methods:**

Mothers (15–25 years old) with infants (< 12 months old) were recruited in person from vaccination clinics in rural Nepal. They were provided with an Android smartphone and a Bluetooth beacon to collect data in four domains: the mother’s location using the Global Positioning System (GPS), physical activity using the phone’s accelerometer, auditory environment using episodic audio recording on the phone, and mother-infant proximity measured with the Bluetooth beacon attached to the infant’s clothing. Feasibility and acceptability were evaluated based on the amount of passive sensing data collected compared to the total amount that could be collected in a 2-week period. Endline qualitative interviews were conducted to understand mothers’ experiences and perceptions of passive data collection.

**Results:**

Of the 782 women approached, 320 met eligibility criteria and 38 mothers (11 depressed, 27 non-depressed) were enrolled. 38 mothers (11 depressed, 27 non-depressed) were enrolled. Across all participants, 5,579 of the hour-long data collection windows had at least one audio recording [mean (*M*) = 57.4% of the total possible hour-long recording windows per participant; median (*Mdn*) = 62.6%], 5,001 activity readings (*M* = 50.6%; *Mdn* = 63.2%), 4,168 proximity readings (*M* = 41.1%; *Mdn* = 47.6%), and 3,482 GPS readings (*M* = 35.4%; *Mdn* = 39.2%). Feasibility challenges were phone battery charging, data usage exceeding prepaid limits, and burden of carrying mobile phones. Acceptability challenges were privacy concerns and lack of family involvement. Overall, families’ understanding of passive sensing and families’ awareness of potential benefits to mothers and infants were the major modifiable factors increasing acceptability and reducing gaps in data collection.

**Conclusion:**

Per sensor type, approximately half of the hour-long collection windows had at least one reading. Feasibility challenges for passive sensing on mobile devices can be addressed by providing alternative phone charging options, reverse billing for the app, and replacing mobile phones with smartwatches. Enhancing acceptability will require greater family involvement and improved communication regarding benefits of passive sensing for psychological interventions and other health services.

*Registration* International Registered Report Identifier (IRRID): DERR1-10.2196/14734

**Supplementary Information:**

The online version contains supplementary material available at 10.1186/s12911-021-01473-2.

## Background

Passive sensing on mobile devices refers to the capture of information that does not require users’ active input while they go about their daily lives [1, 2]. For example, accelerometers on smartphones can detect activities such as walking, riding in a vehicle, and standing, and the Global Positioning System (GPS) captures location. Passive sensors also provide information on the number of steps taken in a day, heart rate variability, exposure to light and sound, and proximity to others with mobile devices. Passive sensing data provides a window onto experiences, behavior, and environments of individuals, all of which are important to understand mental health and mental illness.

Because the field of mental health lacks objective markers of disease such as viral loads, pathogen detection, and point-of-care testing for disease status, passive sensing provides a unique objective reference for mental health status [3, 4]. There are a number of initiatives to explore potential benefits from using passive sensing data in mental health and behavioral health studies. Passive sensing data was collected with people with mental illness in Australia [5]. Passive sensing has been used to identify mood instability [6]. Passive sensing has recorded time away from home and activity levels to identify risk of early dementia [7]. Other studies have similarly explored the potential of using passive sensing in depression [3, 8], bipolar disorder [9], and schizophrenia [10] using GPS location, accelerometers to monitor activity, and various other functions captured by Bluetooth devices. Moreover, unobtrusively collected audio has the potential to reveal vocal biomarkers for depression and other mental illnesses thanks to advances in deep learning and other artificial intelligence applications [11, 12].

Passive sensing data collection can be especially helpful for health initiatives in low- and middle-income countries (LMIC), which are characterized by limited access to specialty health services and where some populations have low literacy [13]. Combined with effective interventions, passive sensing data collection has the potential to address major public mental health issues in LMIC. One mental illness of high prevalence and societal impact is postpartum depression, particularly because young mothers are often not identified or treated in LMIC. The prevalence of postpartum depression in LMIC ranges from 3 to 32% [14]. Passive sensing can help to better understand maternal mental health by recording physical activity, location, sleep, mother–child interaction, and the auditory environment. In a high-income country study of women with perinatal depression, the degree of depressed mood was associated with radius travelled as measured with GPS; women who traveled larger radii had milder depression than the women with severe depression who had smaller travel radii [15]. Given that maternal mental illness is associated with disrupted sleep, lack of social engagement, lack of stability in daily schedules, and altered interaction patterns with their infants [16–18], there are many opportunities to apply passive sensing data collection to improve diagnosis, monitoring, and treatment for mothers with depression.

Passive sensing data collection also has the potential to be a more accurate and less burdensome approach to detection when compared with traditional paper-based screening tools. Typically, self-report checklists—in either paper-based or electronic formats—are used for identification of mothers with depression in low resource settings. Because specialists are often unavailable, tools such as the Edinburgh Postnatal Depression Scale (EPDS) and Patient Health Questionnaire (PHQ-2 and PHQ-9) become de-facto diagnostic tools [19]. These tools typically have high false positive rates (low specificity), which can further burden health systems trying to deliver mental health services in low-resource settings [20]. To additionally complicate the situation, these tools need considerable cultural adaptation to perform appropriately with diverse global populations [21–24].

The strategy of passive sensing using personal mobile phones is a low-cost alternative to traditional screening for identification of mothers with depression because it does not rely solely upon hiring health workers to conduct clinic-based or community screenings. Because an increasing number of mothers now have smartphones, even in low-resource settings, this technology may be cost effective [25]. Passive sensing is also not a time burden or disruption for mothers, in contrast to effort required for actively responding to symptoms checklists and other assessments. Passive sensing does not have the same social desirability bias introduced when responding to a health worker administering a screening tool. Passive sensing is also a real-time process which can provide tailored feedback to both mothers and health workers, as opposed to screening tools that have potential recall bias and provide a generalized overview of experience over the past one to two weeks. Furthermore, passive sensing may have greater sensitivity (fewer false positives) by identifying those persons with an objective impairment in social, behavioral, and physical activities. Passive sensing also illuminates domains a person may have impairment, whereas psychological symptoms on screening questionnaires may not reveal this information.

Before designing a passive sensing-informed intervention program, it is crucial to understand the feasibility of collecting data and cultural factors influencing acceptability [26]. Mobile technology for health (mHealth) use in the real world is not only impacted by technical challenges but also by familiarity with technologies and cultural attitudes and practices [27]. How people interpret the collection of their personal data and the knowledge with which they provide consent to do so are among the key ethical issues highlighted by the U.S. National Advisory Mental Health Council in their report on *Opportunities and Challenges of Developing Information Technologies on Behavioral and Social Science Clinical Research* [28].

Therefore, in this study, we explore the feasibility, acceptability, and perceived utility of collecting passive sensing data among depressed and non-depressed adolescent and young mothers in rural Nepal. We pilot test the passive sensing data collection component of the Sensing Technologies for Maternal Depression Treatment in Low Resource Settings (StandStrong) platform. Specifically, through a smartphone app and Bluetooth Low Energy beacon, sensing data are passively collected on mothers’ geographic movement, physical activity, the audio environment, and mother-infant proximity. The two main sources of data are the amount of passive sensing data recorded and qualitative interviews with mothers about their experience of the passive data collection process. The results reported here are limited to feasibility and acceptability; future publications will present analyses of the content collected and its relation to depression status and treatment outcomes.

## Methods

### Overview

The study protocol is outlined in detail elsewhere [29]: International Registered Report Identifier (IRRID): DERR1-https://doi.org/10.2196/14734. The procedures and results described here refer to Component 2 of the original study protocol. For details on the study according to the EHEALTH extension to CONSORT guidelines [30] see attached Additional file 1: File 1 and RE-AIM framework [31] in Additional file 2: File 2. Recruitment and data collection occurred between November 2018 through April 2019.

In brief, young mothers (15–25 years of age) with infants (< 12 months of age) were recruited from vaccination clinics in rural Nepal. Both depressed and non-depressed young mothers were recruited. The mothers then participated in 2 weeks of passive sensing data collection capturing her physical activity, geographic movement, the auditory environment, and mother’s proximity to her infant. Technologies piloted for passive sensing included Android smartphones, smartwatches, and Bluetooth Low Energy beacons.

### Setting

The study was conducted in a setting that exemplifies limited health resources. This site was Chitwan district, a southern region of Nepal. The total population of Chitwan is 579,984. The under 5 mortality rates for Chitwan is 38.6 per 1,000. The literacy rate is 78.9%, with considerable gender disparities because fewer girls are sent to and complete schooling [32]. Chitwan district was selected because of a longstanding established partnership with the local health system and a district-wide scaling-up of community-based mental health services that was being conducted [33].

### Study population and sampling

Study participants were young mothers (15–25 years old) with infants (< 12 months), including both mothers with and without postpartum depression. Recruitment of mothers was conducted at infant immunization camps held at seven health facilities in rural areas of Chitwan. Camps were typically attended by 136 mothers on average every month. Inclusion criteria were mothers between 15 and 25 years of age with an infant aged between 1 and 12 months living in the study area, and willing to be screened for postnatal depression. There were no inclusion requirements for computer/internet literacy on the part of mothers because of the passive nature of the mobile sensing data collection. The intention was to assess the feasibility among representative mothers in the community, which includes women with limited technology literacy.

To determine eligibility, trained research assistants approached mothers at immunization to ask their age and infant’s age, after which they conducted the consent procedures. For mothers 15–17 years old, assent was obtained, and a guardian provided consent. Because of passive sensing data collection that captured information about the household, a meeting was held with the mother’s household representative to describe the study and data collection procedures. If mothers and their family members agreed to the study, passive sensing data were collected for 2 weeks (14 consecutive days; details on passive sensing described below).

The age range of 15–25 years was selected because this is based on the United Nations definition of youth which includes 15–24 years of age [34]. Similarly, the lower age limit of 15 years old was used because data are routinely collected on pregnancy for mothers 15 years of age and above, such as is in the Nepal Demographic Health Survey (DHS). The women’s module of UNICEF’s Multiple Indicator Cluster Surveys (MICS) also collects data beginning at 15 years of age [35]. We included 25 years of age as well because of prior research on suicide deaths in Nepal which showed the greatest burden of suicide mortality among women was ≤ 25 years of age [36].

A sample size calculation was not conducted because this is a pilot study and the recruitment was done based on feasibility of using these devices by the participants [37, 38]. Our goal was to recruit 25 depressed and 25 non-depressed mothers. To allow for potential dropouts, we allowed for recruitment of up to 27 consenting mothers in each category. We enrolled more mothers than our target because we anticipated dropouts due to novelty of the study and potential reluctance from the participants in using the technology. This sample was based on feasibility of the number of mothers in the youth age range attending local clinics during the study period.

### Mental health measure

Mothers depression status was determined with the Patient Health Questionnaire (PHQ-9), which is 9-item self-report tool for depression screening widely used in both high-income countries and LMIC [39]. The PHQ-9 has been validated for use in Nepal [21]. We categorized the mothers as “depressed” and “non-depressed” based on their PHQ-9 total score. For the purposes of this study, mothers scoring below 9 were classified as ‘non-depressed,’ and those with a score of 9 or above as ‘depressed’. Among Nepali adults presenting to outpatient services, a cut-off of 9 has a sensitivity of 94% and specificity of 69%, positive predictive value (PPV) of 0.33 and negative predictive value (NPV) of 0.99. For non-depressed, we used a cut-off of less than or equal to 7, which has a sensitivity of 100%, specificity of 55%, PPV of 0.26, and NPV of 1.00; this means that an adult presenting to primary care centers with a score of 7 or below has an extremely low probability of having depression (i.e., low probability of being a ‘false negative’). Of note, specific psychometric values for Nepali postpartum mothers aged 15–25 are not available. Additional details on study measures are available in the published protocol [29].

### Technology and passive sensor data collection

Passive sensing was collected through two devices: mothers were given a low-cost *Android Samsung J2 Ace smartphone* and a *Bluetooth Low Energy beacon* to be attached to her infant’s clothing. The devices used in this study were selected following extensive ethnographic inquiry regarding acceptability and feasibility in the study site [26]. The two devices selected for this study (smartphone and Bluetooth beacon) were considered culturally acceptable and feasible based on that formative work. The Samsung J2 Ace smartphone is a mobile phone (US $160) that is popular in the study setting. We selected the Samsung J2 Ace phone because it is widely available for purchase within Nepal and it was the cheapest option that could effectively run all the features and apps required for the study. Common, low-end mobile phones in Nepal cost US $70-$120, and therefore the device selected in the study was slightly more expensive than commonly-used devices. In the study area, most individuals owned mobile phones or have family members who own mobile phones. Hence, there is limited risk of stigmatization because of phone use in the study.

For a subset of four mothers, we also piloted the use of *smartwatches* in place of the smartphone. Models included the Zeblaze Thor 4 Android smartwatch and Lemfo Lem8 Android smartwatch. The cost of the smartwatches was approximately $200. The smartwatches are not yet commonly used compared to the mobile phones but they have the potential of providing the same data in a more convenient device. We gave it only to the subset of the mothers as an exploratory component of the study that was added after the original design and implementation commenced.

The Bluetooth Low Energy beacon was the RadBeacon dot ($10–15) developed by Radius Networks [40]. We used a closed cloth pouch (Nepali: *thaili*) to hold the beacon around the baby’s waist and prevent the infant from being able to remove or play with the device. Because the beacon was sewn into the pouch around the waist, the baby could not access the beacons, or get hold of the device. The research assistants informed the mothers about the safe use of beacons, including caution while using the device, so the device would not cause physical discomfort to the baby. The use of the RadBeacon Dot was approved by the Nepal Health Research Council for the purpose of this study.

The RadBeacon Dot also has United States Federal Communications Commission (FCC) certification. FCC is a body that oversees the permissible exposure level for all devices with radio frequency. In the United States, the Food and Drug Administration (FDA) relies on FCC for inputs on medical devices [41]. According to FDA, devices such as activity trackers are general wellness devices because these devices have “(1) an intended use that relates to maintaining or encouraging a general state of health or a healthy activity or (2) an intended use that relates the role of healthy lifestyle with helping to reduce the risk or impact of certain chronic diseases or conditions and where it is well understood and accepted that healthy lifestyle choices may play an important role in health outcomes for the disease or condition,” [42].

Additionally, in our study, RadBeacon is not a medical device used for treatment or for the transmission of health information (e.g., temperature, pulse, respiration) from the infant. It is only used to track proximity between mother and infant during daytime hours. Regarding the safety of exposure for infants, the FCC limit for radiation from devices is 1600 mW/kg [43], which equates to approximately 800 mW for a 5 kg infant. The RadBeacon Dot specifications are + 4 to − 20 dBm, which equates to 2.5 to 0.1 mW. These ranges are comparable to an infant in a house with a standard wireless network and Bluetooth devices.

**Fig. 1:**
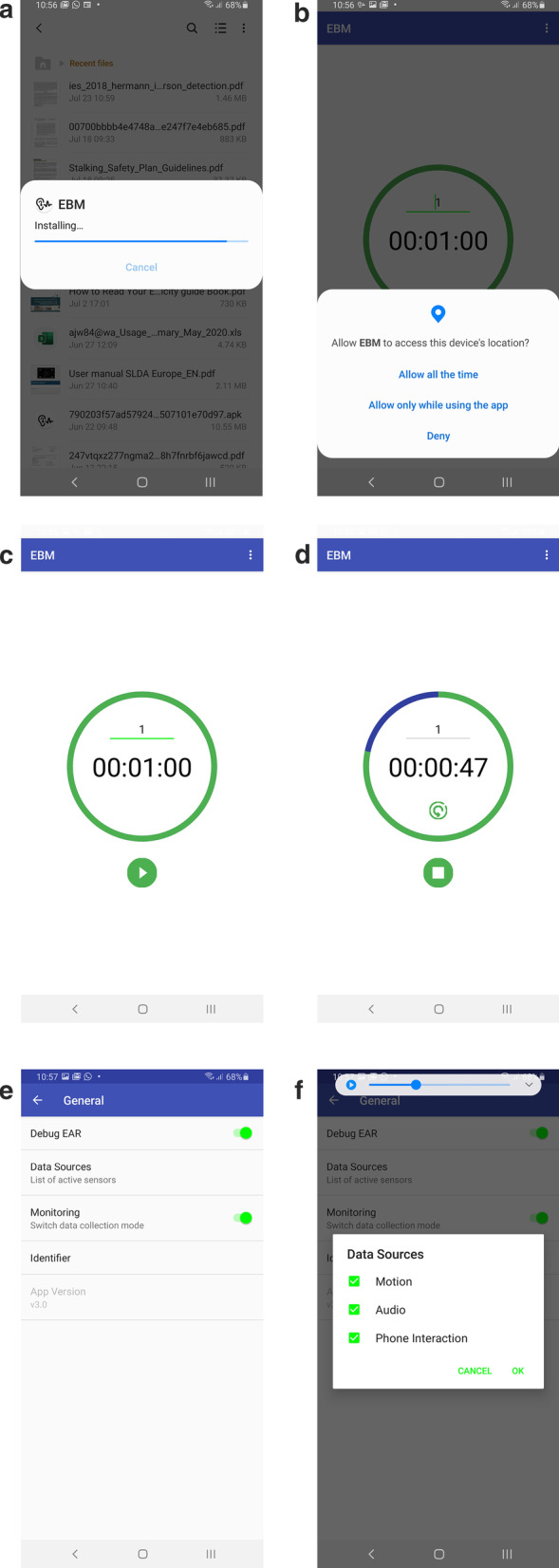
Screenshots of Electronic Behavior Monitoring app (EBM version 2.0). **a** EBM package installer; **b** EBM permission controller; **c** EBM privacy timer; **d** EBM privacy timer running; **e** EBM settings; **f** EBM settings sources

The mothers were provided with the phone and beacon for the duration of the study. They returned the devices after completing data collection. The smart devices collected 4 types of data—*proximity, episodic audio, physical activity,* and *geographic location*. To collect these data, we installed our custom-built Electronic Behavior Monitoring app (EBM version 2.0). The EBM app passively collected data for 30 s every 15 min between 4:00AM and 9:59PM (i.e., 18-h intervals of data collection per day). The EBM app starts automatically whenever the device is turned on. A folder, NAMASTE, was created automatically once the EBM app was downloaded on the smartphone (see EBM screenshots in Fig. 1). All data were stored in the folder. Because all sensing and data collection was done passively, mothers did not need to interact with the app in any way to enable data collection. Details of each passive sensing domain are provided below:

1. *Proximity of mother to infant* The proximity sensor (Bluetooth Low Energy beacon: RadBeacon Dot) was fitted to the infant’s clothing, and the mother was asked to carry the mobile phone to record when the mother was in proximity to the infant. Every 15 min, the EBM app scanned for the presence of advertising packets from the assigned Bluetooth beacon and recorded whether the beacon was present or not. Through the proximity beacon, we calculated the daily interaction routine between mother and child. Routinization of daily behavior is associated with positive mood, less fatigue, and lower risk of maternal depression [16, 44]. We hypothesized that non-depressed mothers will have a more consistent routine of infant interaction than depressed mothers over the 2-week period. Additionally, we anticipated depressed mothers to have less self-care time, and less social support to take care of the baby. Lack of social support is correlated to risk and severity of postpartum depression [17, 45, 46], while high levels of instrumental social support are associated with lower postpartum depression symptom severity [47].
2. *Episodic audio recording* For episodic audio recording conducted approximately every 15 min, the microphone in the phone was used to record 30-s audio clips saved in an .m4a format. The audio data were saved in a local folder on the device before being uploaded to the cloud and processed. We analyzed the audio data by categorizing audio clips as “speech” and “non-speech” sounds. Full details on the audio processing for this project have been previously published [48]. Depressed mothers are more likely to have prolonged periods without verbal communication compared with non-depressed mothers. Human speech sounds, as a proxy for social interaction, helps us measure social isolation which is associated with postpartum depression [49]. Also, lack of social group membership is also considered a risk of postpartum depression [50] especially for adolescent mothers. Limited verbal engagement between mothers and infants is also a manifestation of postnatal depression and predicts poor development for children [51, 52].
3. *Physical activity* Activity recognition was used to record the predicted activity type (e.g., walking, standing still, cycling, riding a vehicle) at the time of audio recording based on the mobile phone’s accelerometer data. In this study, we used the accelerometer and gyroscope sensors along with the Android Activity Recognition API, which is built on top of these sensors. The Activity Recognition API automatically detects activities such as walking, running, riding in a vehicle, or standing still. Self-reported physical limitations are correlated with postpartum depression severity [17]. Wrist actigraphy measurements among postpartum women show an association between disrupted routines and poor mental health outcomes; postpartum women with dysrhythmic fatigue patterns report more stress and less vigor compared with the women where fatigue patterns follow consistent daily cycles [44].
4. *Geographic location* GPS on the mobile phone collected the mother’s position each time the phone had an activity event (phone unlocking, Bluetooth scanning, etc.). We included GPS domain as studies have shown associations between GPS radii and depression [15]: an increase in depressive symptoms predicts smaller radii of travel in subsequent days.

### Explanation of passive sensor data collection to mothers and families

During the consent process with mothers and families, the research assistants explained the various components of the passive data collection, how the information would be used, and demonstrated using the technology. The research assistants explained that this was an initial study to learn about collecting this information and that study would inform how this information could be used in the future to improve health interventions for mothers and infants for mental and physical health. The research assistant demonstrated the proximity beacon information to record when mothers and infants were together. The research assistants explained that the study was not advocating that mothers and infants should or should not be together but rather the goal was to learn about these patterns in their community. The GPS was demonstrated to show how the study would learn about where the mother went in her daily activities. The research assistants explained that the study was not interested in specific locations but rather how much a mother traveled around outside of the house. Again, the research assistants explained that travel outside the house was not considered good or bad for mother’s and infant’s health but rather it was something that the team wanted to learn about for different mothers. Similarly, the activity data was explained as capturing when mothers were resting or physically active. This also was framed as not recommending that mothers rest or be active but rather to learn about what mother’s patterns were. Finally, the audio recording was demonstrated. The research assistants clearly stated that the team was not interested in what mothers and family members said. Instead, the focus was to learn how much of the time there was speaking around the mother, as well as other sounds such as cleaning and cooking activities, vehicle sounds, animal sounds, and other things the recordings would capture. The research assistants explained that the recordings would be used to teach computer programs to better distinguish among these sounds so that future health interventions could use this type of information to improve care for women, infants, and families. All mothers and families were given an opportunity to ask questions about the mobile data collection and data usage. In addition, research assistants followed up with mothers and families during subsequent home visits as additional questions arouse.

Anticipating the privacy concerns, we had a “privacy timer” in the EBM app that allowed mothers to pause the app for as long as they wanted. Once the privacy timer was on, the EBM app stopped collecting data until the timer was closed. In addition, strategies to maintain confidentiality, such as deleting audio files was piloted with mothers using similar devices in South Africa [53]. On the devices, mothers can delete audio files at any time. They can also ask the research assistants to not transfer the data if they do not wish to share it with the team. Mothers were also instructed to turn off their phone anytime they chose in order to stop data collection. Because of our prior work piloting passive sensing technologies and evaluating mothers’ ability to delete data [26, 53], we did not conduct further formal usability testing with mothers for this activity within the current study.

A female research assistant briefed each mother and her family on the technical use of the phone and beacon. All research assistants self-identified as employees of the non-governmental organization Transcultural Psychosocial Organization (TPO) Nepal. The research assistant visited the mother’s home on average 3–5 times over the two-week period, which included study briefing and collecting consent, day one of data collection for technology delivery and training, day three of data collection for technology troubleshooting, and then weekly, with intermittent phone calls to additionally troubleshoot and provide any needed technology support. Mothers were instructed to keep their mobile phones with them as much as possible and attach the beacon to their infant’s clothing throughout the day. Mothers were asked to turn the mobile phones off and remove the beacon from the child during the night. Mothers’ identifiable information (name, phone numbers) were stored in a secure server. The app was password protected, with counselors needing a passcode to access the app. All the passive sensing data and qualitative data were stored under a unique participant code (without identifying information) and stored in a secure cloud-based server. Mothers and family members had the opportunity to become comfortable with the researcher assistants because of their repeated visits to participants’ homes. We had previously produced a video to explain these data collection processes to potential study participants [26].

No prompts or reminders were provided to mothers electronically once the EBM was installed. Research assistants visiting the home would check the functioning of EBM app and detection of the Bluetooth beacon, then they would conduct troubleshooting as needed. Once the mother was comfortable sharing the data, research assistants copied the data from the phones in a portable device. The data did not contain any identifiable information, and contained passive sensing data in .csv and .m4a format. The data folder was coded with a de-identified ID to anonymize the data. It was then uploaded to a secure cloud server, through a secured connection and removed from the local devices within 24 h.

Two types of app errors were collected: app exceptions and user engagement issues. App exceptions and failures can have multiple causes. Commonly they include errors in code logic which can be introduced, for example, when code runs on different versions of Android, or when hardware interfaces are implemented in a non-standard way by device manufacturers. User engagement issues include trouble remembering a password, trying to perform an unsupported function, and/or struggling to find a function. In this study we tracked fatal app exceptions and user-login challenges.

### Qualitative data collection

To assess feasibility and acceptability of passive data collection, we triangulated several sources of data including in-depth interviews (IDIs) performed at the end of 14 days of passive sensing data collection, field notes recorded by research assistants from each participant encounter, and memos documenting the significant events (e.g., drop outs, service outages). Female research assistants conducted IDIs using a semi-structured interview guide lasting between 20 and 45 min. Questions elicited maternal experiences and perceptions of the technology and EBM application, covering feasibility, social acceptability, confidentiality, utility, and recommendations for improvement. Our inquiry focused on confidentiality and social acceptability given important ethical considerations of passive data collection. Questions regarding these domains were elicited both from the mother as well as from her family throughout the study period. Importantly, the research assistant had established meaningful rapport with both the mother and her family (on average visiting the mother’s home 3–5 times), permitting more comfort and allowing detailed and frequent field notes to capture examples and texture not captured by the IDI, as well as notes related to confidentiality concerns from either the mother or her family members. Qualitative interviews were audio-taped, transcribed, and translated before coding and analysis. The interviews were first transcribed verbatim in Nepali and then translated to English by a bilingual translator. We followed a standardized Nepali mental health glossary for translation of emotional and psychological terms into English [54]. The Consolidate Criteria for Reporting Qualitative Studies (COREQ) checklist is included in Additional file 3: File 3 [55].

## Data analysis

### Passive sensing data analysis

Through the EBM v2.0 app, the proximity, activity, and GPS were captured and stored in the comma separated file (Flat file) and the audios were captured as .m4a audio files. Raw data retrieved from the sensors using the EBM v2.0 app were saved on the device with one file being produced per day. The time stamped data were then extracted from these csv files before being processed and loaded into a SQL database. The same approach was followed for each of the study devices resulting in a single aggregated data set per sensor, i.e., proximity, activity, audio, and GPS. During this process raw proximity data were turned into an hourly binary reading indicating the presence or absence of a beacon. Raw activity data were trimmed from the full list of activities detected along with their probability scores to only the activity with the highest associated probability. The 30-s audio wav files were processed by our machine learning model and the audio classification label with the highest probability score saved. GPS data were also dichotomized indicating either the presence or absence of a GPS reading with an accuracy reading of less than 50 m. These four datasets were then loaded into Python where they were merged into a single data frame with a datetime index. Whenever a sensor had no hourly reading it was coded as missing. This data frame was then cleaned to remove data of mothers who dropped out of the study, and fix outliers introduced by some readings missing a valid datetime value. Exploratory data analysis was performed on this data frame including the calculation of measures of central tendency, range and missing value analysis.

Analyses were conducted on the full available daily dataset (4:00AM to 9:59PM) for approximately two weeks of data collection per participant. For the current analyses, collected data are presented in terms of hour-windows. For example, any hour with at least one proximity reading (either present or absent) is considered a ‘collected window’. However, the hour may contain up to 4 readings (e.g., every 15 min). This was similar for audio collection, GPS, and activity. They may have had more multiple readings in an hour, but here we focus on whether there was at least one reading of that particular sensor in an hour window. In summary, the maximum total number of proximity readings in a day would be 18 h × 4 readings per hour (72 readings per day), but we are focusing on 18-h windows that contain at least one reading (i.e., a maximum 18 h per day when at least one reading could be collected). This is because for future functionality of the app, we anticipate counselors will need at least one reading per hour for the information to be useful to inform clinical care.

### Qualitative data analysis

In our prior formative study, we categorized feasibility and acceptability of wearable technology for passive sensing data collection for health research in LMIC into six domains [26]. We incorporated these for the current study, exploring domains relevant to our data including technical issues, interference, confidentiality, safety, utility, and communication. Interview transcripts were combined with their relevant field notes, providing additional context. Three researchers (AH, AP, SM) independently read two transcripts each to generate common themes. SM generated a preliminary codebook. AH, AP, and SM then modified and iterated the codebook during the process of obtaining intercoder agreement. Intercoder agreement was assessed between all coders on a portion of coded data until a kappa of 0.76 was achieved. Three researchers (SM, AT, CI) coded the interview transcripts in NVivo 12 [56]. Code summaries were produced for each of the sub-domains following an applied thematic approach [57, 58]. Summaries were discussed, enhanced, and revised with several authors (SM, AT, AP, AH) to ensure depth, breadth, and systematic comparisons across informants. Case memos were independently reviewed and analyzed by SM and AT to identify case studies relevant to the domains of the paper. We did not approach the participants later for interviews who dropped out of the study but we prepared detailed dropout case memos to record the reasons for dropout and related significant information.

### Ethics and participant safety

The study was approved by the Nepal Health Research Council (#327/2018) and George Washington University’s Institutional Review Board (#051845). As a compensation for their time contribution in the study, the mothers received a set of clothes and toys for their children (US$3-5). The implementing research organization, TPO Nepal, had an institutional suicide protocol in place for handling and reporting any kind of adverse event during the study. During screening and assessments, we did an in-depth risk evaluation and the counselor provided immediate support if there were identified risks of suicide. In case of emergency, we had established a referral system with the government hospital for immediate care by the psychiatrist.

## Results

### Recruitment and sample

We screened 782 mothers, of whom 320 were eligible for inclusion based on age criteria of the mothers and infants. We consented the eligible mothers and screened them for depression. Approximately 92% of the eligible mothers scored below the depression cut-off (n = 294) and 8% scored above the cut-off (n = 26). We serially enrolled non-depressed mothers. Approximately two-thirds of the non-depressed mothers and their families provided consent and were enrolled, with a final sample of 27 non-depressed mothers consenting to participate in the passive data collection. All mothers subsequently screened, who scored below the depression cut-off, were not included in the study. Of the 26 mothers who screened positive for depression, 11 of the mothers and their families provided consent for enrollment. Table 1 contains demographic information of the participants. Reasons for not participating included mothers moving outside of the study area, inability of the research team to contact mothers following initial screening in the health facilities, families not-consenting to participate in the study, and mothers too busy to participate due to family obligations. Two participants withdrew from the study before completing the full 2 weeks of passive sensing data collection (additional details on why they withdrew are provided below).

**Table 1:** Demographic characteristics of study sample (n = 38)

Characteristics	N (%)
*Mother’s mental health status*	38 (100)
Depressed	11 (28.9)
Non-depressed	27 (71.1)
*Mother’s age*	
15–18 years	11 (28.9)
19–22 years	22 (57.9)
23–25 years	5 (13.2)
*Mother’s caste/ethnicity*	
Brahman/Chhetri (upper castes)	8 (21.0)
Janajati (ethnic minorities)	18 (47.5)
Dalit (lower castes)	12 (31.5)
*Mother’s religion*	
Hindu	32 (84.2)
Buddhist	3 (7.9)
Christian	3 (7.9)
*Mother’s education*	
Grades 1–5	7 (18.4)
Grades 6–10	24 (63.2)
Grades 11–12	7 (18.4)
*Mother’s occupation*	
Business	3 (7.9)
Housewife	29 (76.3)
Agriculture	4 (10.5)
Day wage laborer	2 (5.3)
*Mother’s number of children*	
One child	31 (81.6)
More than one child	7 (18.4)
*Mother’s smartphone ownership*	
Yes	26 (68.4)
No	12 (31.6)
*Infant’s gender*	
Male	16 (42.1)
Female	22 (57.9)
*Infant’s age*	
1 to 4 months	21 (55.2)
5 to 8 months	10 (26.3)
9 to 12 months	7 (18.5)
*Household: Electricity*	
Yes	36 (94.7)
No	2 (5.3)
*Household: WiFi internet at home*	
Yes	3 (7.9)
No	35 (92.1)

### Passive sensing data collection

We collected activity, audio, GPS and infant proximity data through passive sensor readings every 15 min from 4:00AM to 9:59PM daily. Table 2 provides the hours of total passive data collected between 4:00AM to 9:59PM among depressed mothers, non-depressed mothers, and the total sample. For mothers who participated for exactly 14 days, the total number of hours with readings from 4:00AM to 9:59PM was 252 (14 days * 18 h = 252 h-readings). However, there was variation by start time and end times with some participants participating for more than or fewer than 252 h. Some participants had atypical start times of their passive sensing collection or delayed cessation of their collection based on when research assistants could meet them.

**Table 2:** Passive data collected daily for two weeks from 4:00AM to 9:59PM

Passive sensing domain	Average total possible number of readings per participant	Average observed number of readings collected per participant	Percent mean observed readings per participant	Range of percent readings (min–max)	Median percent readings (IQR)
*All participants (n = 38)*					
Audio	252	144.6	57.4	11.7–97.2	62.6 (25.1)
Activity	252	127.5	50.6	0–95.5	63.2 (51.1)
Proximity	252	103.6	41.1	0–84.2	47.6 (32.5)
GPS	252	89.2	35.4	0–85.3	39.2 (45.4)
*Non-depressed participants (n = 27)*					
Audio	252	145.7	57.8	13.2–87.9	63.7 (17.5)
Activity	252	121.2	48.1	0–87.9	62.4 (53)
Proximity	252	110.6	43.9	1–84.2	51.8 (35.4)
GPS	252	92.5	36.7	0–85.3	40.8 (47.9)
*Depressed participants (n = 11)*					
Audio	252	142.9	56.7	11.8–97.1	51.2 (31.9)
Activity	252	143.4	56.9	0–95.5	64.0 (28.1)
Proximity	252	85.7	34.0	0–67	32.9 (20.8)
GPS	252	81.6	32.4	0–77.5	30.4 (39.9)

**Fig. 2:**
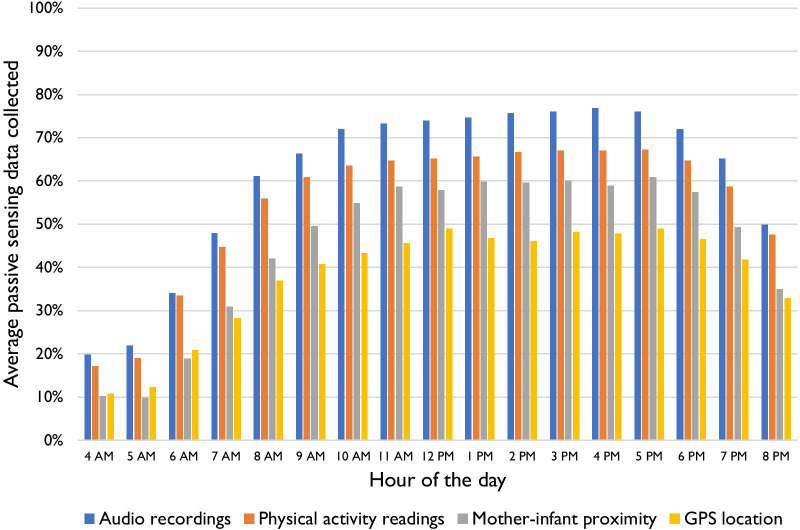
Average passive data collection by the time of day, based on readings collected for two weeks with depressed and non-depressed mothers

Audio and activity data were captured more often than GPS and proximity data on average. Per participant, there were on average 57.4% of hours when at least one audio reading was collected, 50.6% for activity, 41.1% for proximity, and 35.4% for GPS. **Figure ****2** shows the average passive data readings by the time of the day. Data collection was lower than the total possible readings in the early morning across all sensors and tapered off at night, but was generally consistent from 10:00AM to 5:59PM. We explored possible explanations for differences in successful data collection by time of day and sensor type along with description of qualitative results to illuminate these differences.

We categorize these findings below based on the 6 qualitative domains for feasibility and acceptability (see Table 3).

**Table 3:** Qualitative domains

Domains	Definition	Code examples
Domain 1: Technical issues	The degree to which technical issues in the devices impact use or the devices	mobile use, data usage, family involvement
Domain 2: Interference	The degree to which the device may impact physical functioning, activities, or daily routines	mobile use, battery, beacon use
Domain 3: Confidentiality	The degree to which the device would protect personal information	privacy concerns, social perspective
Domain 4: Safety	Perceptions regarding health risks or put a child, mother or family at the risk of mugging or theft. This domain also explores safety concerns mothers have over losing or breaking the device itself	child safety, mother safety, device safety
Domain 5: Utility	The perceived benefits of the device for improving caregiver and child health, development, and mental health	non-study specific utility, study specific utility, misperceptions and other perceptions
Domain 6: Communication	Communicating study objectives and device use to the mothers during the consent process at the beginning of the study and by study team engagement throughout the study duration	autonomy, study team engagement, consent/debriefing process

#### Domain 1: technical feasibility

Mobile phone battery charge, data usage, and positive and negative family involvement were the main technical feasibility issues identified that limited data collection throughout the day. Passive sensing requires the phone to be turned on. Morning data (and some evening data) were most likely to be missing because mothers typically were instructed to turn their phones off at night before bed and then turn it back on when awakening in the morning. Subsequently, there were gaps in data collection in the evening and early morning hours.

*“I used to switch off this watch at night and connect to the charger and switch on in the morning. … In the daytime I used to switch off this watch again and connect it to the charger.”*

Depressed mother (19–22* years, *Note: age ranges are used for all quotes to protect anonymity)

Common feasibility challenges likely reduced data capture across all sensor collection. These feasibility challenges included lack of electricity, mothers forgetting to charge the phone, devices not retaining charge (especially smart watches), technical difficulties such as phones not connecting to the charger properly, or environmental factors such as protecting the device from rain. In general, there were more technical issues reported by mothers who used smartwatches with frequent battery draining. We tested the use of smartwatches instead of mobile phones with four participants but 0% of the activity data was collected due to the smartwatch not supporting activity data collection (see Text Box 1). The use of smartwatches with no activity data contributed to reducing average across all participants to 60% at best, with the greatest data capture during midday.

**Text Box 1:** Limitations of passive sensing data collection with a smartwatch

For a non-depressed mother (19–22 years), it was her first experience of using a smartwatch. She did not have a problem attaching the beacon on the child’s clothing. However, she felt uncomfortable wearing the watch on her wrist. This was largely due to her workload at home – she washed clothes and did dishes multiple times a day, which necessitated her to get her hands and arms wet. She was concerned about possible water damage to the watch. She also had trouble keeping the watch charged because it lost battery quickly. Her husband helped her complete the study, providing reminders and assistance charging the watch. We collected 62.5% and 51.9% audio and proximity data but were unable to collect GPS or activity data due to limited functionality of the smartwatch.	

A likely reason for less successful data capture compared to audio was that GPS data collection was not triggered the same way as audio, proximity, or activity data were. The GPS data collection required WiFi or 3G signal for collection. Therefore, data collection was low because mothers were mostly indoors which limited this GPS connection. Initially, we used a combined method that included both mobile data and direct GPS connection to collect GPS location of the mothers. However, mothers and their families quickly used available data, typically by watching YouTube videos, which then led to not enough prepaid data for GPS collection. We switched to direct connection to the satellite to address the loss of data due to low mobile data by turning on high accuracy in the GPS settings.

#### Domain 2: interference

We explored daily interference related to mobile phone and Bluetooth beacon use. Consistent daily mobile phone use was acceptable to most mothers. However, carrying two mobile phones (one for the study and one personal phone) was considered interfering with daily activities. Additionally, mothers shared their concern about the probability of losing mobile phones if they had to carry two phones at all times. Mothers working in a shop or office outside the home and mothers who were farming found it even more challenging to carry two mobile phones. Additionally, it was difficult for the study team to contact mothers who worked during the day if she had technical issues with the devices, which caused further disruption in data collection (see Text Box 2).

**Text Box 2:** Working mothers and passive data collection

A non-depressed mother (23–25 years) was a small business owner of a shop in which she made toy dolls and trained others in this trade. She received assistance from her family to use the smartwatch. Her husband helped her charge the watch when the battery was low, reminded her to wear the watch, and put the beacon on the child (in the secured pouch). As she was busy with her business’s work, it was difficult for her to have enough free time for regular study team visits. So, she suggested recruiting housewives instead of employed mothers so that they could dedicate their time to the study. She completed the study, and we were able to collect 62.7% of audio and 54.1% of proximity data. (No GPS or activity data were recorded due to technical issues in the smartwatch.)	

The participants who did not find mobile phones interfering typically wore clothes with pockets or used the mobile phones for non-study related activities such as watching videos or listening to music. Bluetooth beacons presented more challenges with daily interference. This may have been due to beacon novelty to the mothers so they felt more concerned initially about the device than about mobile phones. One of the mother’s major concerns was physical discomfort to the baby. Mothers generally put the beacons on the baby and took it off at night, or during oil massages and baths. Some mothers found the routine of putting beacons on the baby tedious after the first few days with some mothers delaying or forgetting to put the beacon on the baby after bathing or oil massages.

The proximity data was consistently lower than both audio and activity data with about 60% data collection at midday, even though it was triggered at the same time as audio and activity. One possible reason could be the participants turned off the Bluetooth signal on their mobile phones.

#### Domain 3: confidentiality

On average, each participant contributed approximately 57.4% of the possible audio from 4:00AM to 9:59PM. Audio recordings were the data type most concerning to the participants from a confidentiality perspective. At least 11 mothers in our study expressed concerns of being audio recorded. One of the reasons was fear that study staff would listen to the audio clips and share private information with other people in the community, or that the community would know about the family disputes. For instance, two participants reported:

*"If I talk about difficulty, mostly I am worried that other people will listen to all our discussions. They will know all our family problems. And they will talk about our problems everywhere."*

Depressed mother (23–25 years)

*Interviewer [I]: Do you like this beacon and this mobile? Do you like to use this mobile and fix this beacon on your baby?*

*Participant [P]: I like using the devices but I feel worried because you might know all our family matters and our conversations.*

*I: We don’t listen to those recordings.*

*P: You will not share those recordings with other people?*

Depressed mother (19–22 years)

Some participants reported changing their behavior such as spending more time with the baby, talking lovingly or softly around the baby, shutting the mobile off during family arguments, or asking family members to not use bad words. Each of these behaviors may have reduced or biased the audio capture. Mothers with family members that abused alcohol and mothers in conflict with their mothers-in-law usually expressed the greatest privacy concerns. Family members of such households also asked mothers to switch their mobile phones off when they were under the influence of alcohol, or delete audio recordings that had their voices (See Textbox 3).

*“We have different conditions in our home. Sometimes people quarrel and we have arguments in our house. This mobile might record all those things so I have to switch off this mobile.”*

Non-depressed mother (15–18 years)

P: [Smiling] *This mobile records sound. And all the recordings were stored in its memory so my husband told me to delete his recordings.*

*I: When did you switch off this mobile at night?*

*P: Sometimes at 6 or sometimes at 7 pm.*

*I: And what about your sisters and your own mother?*

*P: Sometimes when I start talking about my mother-in-law, my mother tells me not to talk about her, this mobile might record the voice and save it.*

Non-depressed mother (15–18 years)

#### Domain 4: Safety concerns

Three types of safety concerns were highlighted by participants: (1) safety of the infant when the Bluetooth beacon was attached to his/her clothing, (2) mother’s safety when using mobile phones, and (3) physical safety of the devices. Among the three, child safety was the most concerning to the mothers. One major child safety issue was concern about possible physical discomfort that the beacon could cause to the infant, such as device poking the baby during sleep.

“*I think that this beacon might poke my baby, and make it difficult for the baby to sleep.”*

Non-depressed mother (19–22 years)

To avoid the beacon causing discomfort, mothers were instructed to remove the beacons when the baby was sleeping or mothers moved the device over multiple layers of clothes to avoid bothering the infant. The feeling of potential discomfort to the child hindered the consistent use of beacons and may have been one factor for reduced proximity data capture.

*“While using these technologies, I thought this beacon might poke my baby and sometimes I removed the beacon.”*

Non-depressed mother (15–18 years)

Despite child safety concerns, there were two major facilitators that propelled mothers to continue using the device: trust in the study staff and no adverse effect to the baby after the first few days of use. Trust in study staff was also supported because mothers had initially met the study staff during recruitment in health posts where mothers went for regular checkups and immunization, and mothers trusted the health workers in the health facility. The study staff coordinated with the health workers and were therefore seen as trustworthy. Second, despite initial concerns, when the baby did not get sick or have adverse effects in the first few days of use, the mothers were reassured that the beacons were safe:

*I: Did you think that it might affect your baby or your baby might feel difficulty due to this beacon?*

*P: In the beginning I had those types of [negative] thoughts but after using [the devices] regularly for many days I didn’t have that type of thought anymore.*

*I: What types of thoughts did you have in the beginning?*

*P: That my baby might get sick, or it might have some health effects.*

Non-depressed mother (23–25 years)

Mother’s safety was less of a concern in comparison to child’s safety. In general, all the mothers thought the mobile phones and beacons did not have an adverse effect on the mothers. A factor facilitating the use of smartphones was mother’s prior experience with mobile phones. Because mothers were familiar with smartphones, they did not think it would affect their health.

**Text Box 3:** Deletion of data and other reasons for low data capture

The family of a depressed mother (19–22 years) lived in a temporary squatter settlement without electricity near the jungle. They agreed to participate, but due to lack of electricity they charged their mobile phones at a neighbor’s house. The participant was concerned that the device may get stolen and worried about needing to cover its expenses. The study team provided her with a power bank to charge the mobile and assured her and her family not to worry if something happened to the device, there would be no financial consequence. The provision of a power bank helped to keep the mobile running for a longer period of time. We collected 73.9% of activity, 41.6% of audio, 10.5% of GPS, and 29.0% of proximity data from the mother. The low GPS data collection was a result of excessive data usage. During data collection, we relied only on mobile data to collect GPS. This and other similar situations where mothers ran out of prepaid data prompted us to change the connectivity so that the phones had direct connection to the satellite. There was lower audio and proximity data collection in comparison to her activity data. In our qualitative interviews, the mother shared that her husband and mother-in-law listened to the audio files and deleted the ones that had their voices. Another reason for her relatively low data capture was that the proximity data collection was interrupted when the Bluetooth was turned off on the phone, which was another reason for the low data collection. The participant’s husband used to turn off the Bluetooth and keep the mobile for his own entertainment purposes due to which proximity data was interrupted.

**Text Box 4:** Religious concerns as a reason for early termination of passive data collection

No participants refused to participate because of religious beliefs with the exception of one family that was concerned that the technology was used for Christian religious conversion. A non-depressed mother (19–22 years old) withdrew from the study after a few days. Through follow up qualitative work, we later learned that the main reason the mother’s family asked her to withdraw was that they suspected that the technological devices were being used to convert them to Christianity (due to a legacy of coercive missionary organizations in the study region). We also learned that the participant wanted to continue the study but was forced to drop out by her husband and father-in-law. We collected 14.5% of activity, 65.5% of audio, 6.3% of GPS, and 7.5% of proximity data from the mother. This mother was also one of the earliest participants we gave the devices to, as we were still making changes to the technology for appropriate data collection. The lower GPS data could be due to the mobile phone running out of data. Other data collection could have been affected by social factors. For example, the mother’s family later told the study team that they were reluctant to use the devices, including the beacon on the child. The lower proximity data collection could mean that the Bluetooth on the mobile phone was switched off most of the time. The higher audio data indicates that the mobile phone was still switched on most of the time, although functionality such as Bluetooth was likely disabled or turned off.	

The final safety concern that we explored was potential theft or breakage of the devices. Mothers, especially those from poor economic backgrounds, were scared that the study devices could get stolen or broken. Despite assurance from the study team that they were not liable in case of theft or accident, mothers were still anxious, especially for the first few days. The study team provided support and reassurance to the mothers during subsequent home visits to assuage remaining anxiety related to device safety.

#### Domain 5: Perceived utility.

In general, mothers and families did agree to starting and then continued using the phones throughout the study period because of perceived benefits. Some of the perceived benefits aligned with the study goals, while some benefits were non-study related. Among the study-aligned perceived utility, mothers mentioned using the beacon and mobile phone to know the distance between them and their babies throughout the days. They could see the frequency of being apart from vs. together with their infants. They also knew the phone recorded their sounds, movement, and activities. Some mothers went back and listened to their audio clips. For perceived utility not related to the study purpose, mothers reported that they used the phone for listening to music, taking pictures and videos, using Facebook and watching YouTube videos. Perceived utility, however, varied across participants. For example, when asked about the utility of the beacons, some mothers said that the study showed how much they loved their babies. Other participants speculated that the data could help understand growth and brain development of the baby, or help in conflict resolution at home.

*I: I gave you this watch and fixed this beacon on your baby for two weeks. How was your experience these two weeks? What was your experience while using this watch and beacon? Please share something about that.*

*P: I think I got a chance to provide more care to my baby. I got a chance to learn many things from the technologies that you provided me. This watch helps to find out whether we are speaking the truth or not. This watch records our voices continuously for a long time. One thought is continuously stuck in my heart-mind: through these recordings we can find out if someone is hiding something.*

Non-depressed mother (19–22 years)

Some mothers thought the technology could record the time spent with the baby or identify their mood changes during the day.

*I:* *Do you know anything about why you are using this beacon and mobile? Though I had already told you about its use, what do you think about this technology?*

*P: These technologies are for observing the changes in a mother like being irritated, distance between mother and the baby and problems in family relations. In the future, our daughter-in-law’s granddaughters would benefit from this technology. That’s why I agreed to use this technology.*

Depressed mother (23–25 years)

#### Domain 6: Communication

Communication facilitated passive data collection through three subdomains: study team engagement, tech literacy, and autonomy of using devices. The importance of the study team engagement was critical when explaining the technology and addressing any queries that mothers had during the study duration. Mothers enjoyed their interactions with the study staff, especially when the study staff asked them about their children and family. They enjoyed study staff visiting them every few days to discuss any new queries and talk to the family members about the technology. During the consent process, we also provided the mothers with a study brief handout in Nepali, as a support tool, so that other family members and neighbors could read and understand about the study. The study handout supported mothers in answering family’s or neighbors’ questions about the study when the study team was not physically present to answer those questions.

One of the major social influences for acceptability was collaboration with local health posts for screening and recruitment at the community level. The recommendation from the health workers helped the study team to establish rapport with the participant and then follow up through the home visits. With the study team’s consistent technical support and clear communication of the study findings, the mothers felt more involved in the data collection process. They also felt more empowered to censor data collection by turning off the mobile device or beacon if needed. For example, 11 mothers described that they switched the phones off or left the phone in another room during family discussions, particularly to avoid recording any disputes or bad language.

Although 32% of study mothers were new to smartphone technology, all mothers confidently described their ability to navigate and operate the varying features by the second week. Mothers made decisions when or whether to attach the beacons on the child’s clothing as well. To support autonomy of using the devices, we found family engagement and consent to be important facilitators in both the research process and successful implementation of passive data sensing (see Text Box 4). Mothers felt more confident and comfortable when the study team explained the technology and study objectives to their families, especially to the family members in decision making roles such as husbands and mothers-in-law. As per our protocol, the study team visited the mother’s family after the initial screening at the vaccination clinics. Family consent helped the family understand the technology better and ask questions to the study team. Mothers generally said they were able to answer questions on the study objectives independently, but the family consent helped them get support from family members when the mothers had to explain the technology to non-family members.

## Discussion

This study collected passive sensing data from 38 young mothers (27 non-depressed and 11 depressed) in rural Nepal. We found that approximately half to two-thirds of mothers approached provided individual and family consent. On average, 57.4% of the hours intervals of data collection had at least one audio recording, as well as 50.6% for activity, 41.1% for proximity, and 35.4% for GPS. As a point of comparison, in a study of passive sensing conducted in a high resource setting (Australia) with 32 people with mental illness, 55% of the possible passive scan readings were obtained [5]. Another study used 50% of possible passive sensing readings per participant as a cut-off for analysis [3]. These studies and others using passive sensing data have encountered similar technological, privacy, and other challenges as we have described in this low-resource setting [3, 8–10, 59].

In our study, positive and negative family engagement, perceived benefits of passive data collection, privacy issues, and technical limitations (such as battery and data usage) influenced the amount of passive data collected. Given the tremendous emerging potential of digital technology to shed light on human behavior and unlock new, and improved, avenues for treating previously intractable mental health problems [28], this study evidences a first attempt to collect such information from a vulnerable target population and makes specific recommendations to optimize subsequent feasibility and acceptability.

Although the most common reason for mothers and families not participating in the study was logistical (e.g., planned travel out of the region to stay with other family members), there were also some families who were unwilling to let the mothers participate. In particular, fewer mothers with depression consented and enrolled. This was due to reluctance in regard to family consent. This is potentially because depressed mothers may be more likely to be in families where a husband or other members abuse alcohol, had other behaviors that were socially stigmatized, or raised safety concerns. Previous studies have reported perceived stigma as a barrier to participation among participants with mental health conditions [60–62]. This could also be the case for the depressed participants in our study. Positive family involvement in the study, especially in cases of severe mental health illness, has been recommended as vital to successful implementation [63]. Thus, future studies may increase feasibility and acceptability by concerted efforts to engage the whole family, not just the household member with a mental illness. For a number of the mothers and their families, they were unclear about the purpose of data collection. This raises the opportunity to improve participation by providing more comprehensive explanations of how this technology will improve health services, especially when conducting exploratory studies using advanced technology in LMIC.

After mothers began participating in the passive data collection, their main concern and that of their families appeared to be audio recording and the potential breaches of privacy from these recordings. Mothers used strategies such as turning off the phone, deleting data, or leaving the phone in a different room to address these confidentiality concerns. Future options to promote privacy when dealing with audio files are processing the audio recordings immediately on the smartphone so we only get text files and scrambling audio so that the speech is unrecognizable when played back [59, 64, 65]. We are currently developing a version of our model that runs on low power edge devices such as phones, which allows the audio to be processed locally, so we will not have to upload them to a server. We would only get the audio classification as a text file. The current study was an initial effort to determine feasibility, which will be a basis for us to improve audio analysis in later studies. Based on analysis of the audio findings from this study, we found that 43% of all passively recorded audio clips were human speech [48]. However, a manual validation found that the machine learning used for audio classification under-detected the frequency of human speech. For future studies, we recommend a culturally tailored machine learning model to distinguish more detailed social sounds such as child laughing, child crying, adult laughter, yelling, etc., which can be important predictors of mothers’ positive and negative social environment [48]. This will also require researchers to address privacy challenges raised by some of the participants in our study, especially if we are extracting more nuanced details of mothers’ social environment.

In addition, refining the consent process with regard to how audio storage is explained is integral for privacy and confidentiality assurance. A clear and more importantly, simple explanation regarding how the audio data will be processed, stored, and analyzed needs to be given to the mother and their family members. This description should not merely be limited to the consent process, but should be available at all times during the data collection. Mid-way through our study, we realized a simplified paper version of this description helped mothers understand and explain the study to others. For future use of this technology, we will also add more video explanations and demonstrations during the consent process so mothers and families have adequate information to understand how privacy is maintained so they can make an informed decision to participate in passive sensing.

Another strategy is to have multiple family members participate in the passive data collection. The EBM app could be installed on husbands’ and other family members’ phones so that they also see the potential benefits of the data collection. Given that postpartum depression often involves relationship stressors among parents and other relatives, male involvement could both improve acceptability of passive sensing data collection and enhance the outcomes of psychological interventions. This is consistent with recent calls for more family involvement in global mental health initiatives given the importance of the family in care and recovery in LMICs [66, 67].

In future studies, technical shortfalls for passive data collection (e.g., limitations in data, battery) could potentially be addressed by using reverse data billing in which the research organization or health organization is billed for data collection through a post-paid contract rather than needing to provide mothers repeatedly with charge cards for data and airtime. For example, the EBM app and other StandStrong apps could be billed to the organization while charges for calls, texts, YouTube, and other personal social use are billed to the mother. In addition, installing the StandStrong suite on participant’s personal phones rather than giving an extra study phone could reduce the challenges related to carrying two phones and help with the learning curve because mothers are already familiar with their own phones [68]. Use of one’s own personal smartphone is common for smartphone-based interventions in high income settings.

We piloted smartwatches as another approach to passively collect data. Because smartwatches can be worn and do not require pockets or purses, we anticipated more acceptability and ease of use for mothers. However, there were a number of limitations. At this stage in smartwatch technology, we found that affordable watches had limited functionality compared to Android smartphones in the same price range. While the EBM app could be used in smartwatches, GPS data were collected sporadically and the accelerometer sensor did not work in smartwatches. Thus, we had to drop the smartwatches from our studies after a few participants’ use. In the future, smartwatches may have increased functionality to allow for these devices to more feasibility collect the needed information compared to carrying around multiple phones [69, 70].

Ultimately, this study is an important first step for the integration of passive sensing data collection into health services for psychological interventions and other health care. We found that approximately 50% of data collection was achieved. This may be sufficient to inform some health services. Recent studies have shown the potential of using passive sensing data to predict depressive symptoms [71–76], and have successfully integrated proxies of social and physical behavior as collected from smartphone sensor data into behavioral change intervention[77]. When incorporated as a part of psychological services, these behavioral patterns can be important in tracking and supporting positive behavior. Our study aimed to determine if collecting passive sensing data was feasible in a rural setting, and what challenges could impact successful data collection. The most important contribution of this work is highlighting the importance of clear and transparent communication with both young mothers and their families about the purpose and process of passive data collection. It is vital to assure that use of such technology does not increase risk of harm for mothers in vulnerable situations.

In a subsequent study component, we will assess validity of the passive data calculating correlations across measures such as PHQ-9 and the ability of passive sensing to distinguish between depressed and non-depressed mothers. In addition, this study sets the groundwork for incorporating passive sensing data into a psychological intervention. In the research site, a psychological intervention was being delivered to support maternal mental health. The intervention, the Healthy Activity Program (HAP), is an evidence-based psychological treatment originally developed in India for delivery by lay counselors [78]. It has also shown promise in reducing the symptoms of depression in Nepal [33]. However, in HAP, counselors rely on self-report to understand a client’s behavioral patterns between sessions. Passive sensing data collection visualized in the StandStrong app for counselors if integrated into HAP has the potential to provide counselors with information related to the activity patterns, sleep, social interactions, and mood of the mothers [79]. Incorporating passive data into HAP can aid counselors to provide personalized treatment. Our ultimate goal—after establishing feasibility and acceptability of passive sensing data collection as described in the current study—is to use passive sensing data collection to inform HAP delivery in Nepal [29].

## Limitations

This study has a number of limitations that should qualify generalizability of the findings. The data collection strategies were evolving over the course of the study. For example, early in the study, we tried introducing smartwatches and then abandoned this strategy because of both limitations in which sensors worked and fears of mothers (e.g., getting the watches wet). Similarly, we changed the GPS data collection strategy midway through the study to increase data capture. Research assistants modified how they explained the study to mothers and families as we gained experience of the collection process. The reported data-capture rates mix together low collection rates from the beginning of the study and higher rates toward the end of the study. The collection rates include two participants who withdrew a few days into the collection process. Our collection rates are conservative estimates of what could be achieved in any studies going forward. Also, the technological literacy and technology landscape are rapidly changing. There is increasing availability of cellular networks in rural Nepal and rural residents are becoming more familiar with smartphones. We anticipate fewer technological barriers to this type of mHealth initiative in the future. However, attending to the social dynamics and daily patterns of phone use will be crucial for successful implementation of mHealth and passive data-augmented interventions in the future.

## Conclusion

This study identified a number of technological barriers and facilitators to comprehensive passive data collection in a rural area of Nepal. Most of these technological barriers can be addressed. More importantly, we identified concerns related to confidentiality and interpretation of the passive sensing data collection. Passive sensing data collection has the potential to transform psychological treatments and other mental health services. Just as glucose monitoring, remote blood pressure monitoring, and other remote approaches to assessing health in real-time, real-world situations, passive sensing data can provide an as yet untapped glimpse into real world behavior and environment. However, to scale use of passive sensing data collection, the approach needs to be feasible and culturally acceptable to potential participants and their families in mHealth programs. Successful implementation of StandStrong and similar passive data collection initiatives will require addressing these concerns and fully involving families in mHealth initiatives.

## Supplementary Information

**Additional file 1.** EHEALTH CONSORT extension checklist.

**Additional file 2**. RE-AIM framework.

**Additional file 3.** Consolidated criteria for reporting qualitative studies (COREQ).

## Data Availability

Data will be made publicly available upon publication of the final study results.

## Abbreviations

EBM: Electronic Behavior Monitoring
EPDS: Edinburgh Postnatal Depression Scale
FDA: Food and Drug Administration
GPS: Global Positioning System
HAP: Healthy Activity Program
LMIC: Low- and Middle-Income Countries
MICS: Multiple Indicator Cluster Surveys
NDHS: Nepal Demographic Health Survey
NGO: Non-Governmental Organization
PHQ-9: Patient Health Questionnaire
PPV: Positive Predictive Value
StandStrong: Sensing Technologies for Maternal Depression Treatment in Low Resource Settings
TPO: Transcultural Psychosocial Organization
UNICEF: United Nations Children’s Fund
