# Supplementary material for: Passive sensing on mobile devices to improve mental health services with adolescent and young mothers in low-resource settings: the role of families in feasibility and acceptability
Source: BMC Med Inform Decis Mak. 2021 Apr 7;21:117. doi: 10.1186/s12911-021-01473-2 (PMC8025381; doi:10.1186/s12911-021-01473-2)
Supplement: Supplementary file 2 — Additional file 2. RE-AIM framework. [file 12911_2021_1473_MOESM2_ESM.docx]

**Supplemental File 2.** RE-AIM Elements by Dimension and Evaluation Criteria (criteria adapted from Gaglio et al.[1])

| **Dimension Evaluation Criteria** | | **Data and analysis** |  |
| --- | --- | --- | --- |
| **Reach** | | | |
|  | Exclusion criteria | 782 mothers screened at infant vaccination clinics; 320 (40.9%) met age inclusion criteria for mother (15-25 years old) and infant (<12 months old) |  |
|  | Percentage who participate | 66% of non-depressed mothers consented among those offered the opportunity to participate in the passive data collection; 42% of eligible depressed mothers consented to participate |  |
|  | Characteristics of participants and non-participants | Qualitative findings suggested reasons for non-participation of both non-depressed and depressed mothers. Reasons for not participating included mothers moving outside of the study area, inability of the research team to contact mothers following initial screening in the health facilities, families not-consenting to participate in the study, and mothers too busy to participate due to family obligations. |  |
| **Effectiveness** *(Note: no measure of effectiveness was a component of this analysis, instead, passive sensing data collection is described in the section below)* | | | |
|  | Measure of primary outcome | Passive sensing data collection for proximity, activity, geographic movement, and audio environment |  |
|  | Measure of primary outcome relative to public health goal | Passive sensing data are hypothesized to associate with mental health status |  |
|  | Measure of broader outcomes or use of multiple criteria | *Not included in the current analyses* |  |
|  | Measure of robustness across subgroups | *Not included in the current analyses* |  |
|  | Measure of short-term attrition | Two participants withdrew from the study before completing the full 2 weeks of passive sensing data collection. One withdrew because of fear the technology was being used for religious conversion. Another withdrew because of family pressure to no longer participate. |  |
| **Adoption-setting level** (*Note: not described in the current analysis)* | | | |
| **Adoption-staff level** (*Note: not described in the current analysis)* | | | |
| **Implementation** *(Note: for the purposes of the current analysis, implementation refers to use of the passive sensing data collection and does not refer to the psychological intervention studied in subsequent phases of the StandStrong study)* | | | |
|  | Percent of perfect delivery | No participants had 100% data collection across all domains of passive sensing data |  |
|  | Adaptations made to intervention | GPS data collection was modified; high data usage apps (e.g., YouTube) were initially turned off on study phones |  |
|  | Cost of intervention: time | *Not recorded in current study* |  |
|  | Cost of intervention: money | *Not recorded in current study* |  |
|  | Consistency of implementation across subgroups | *Subgroup analyses not conducted.* |  |
| **Maintenance-individual level** (*Note: not described in the current analysis)* | | | |
| **Maintenance-setting level** (*Note: not described in the current analysis)* | | | |

1. Gaglio B, Shoup JA, Glasgow RE: **The RE-AIM Framework: A Systematic Review of Use Over Time**. *American Journal of Public Health* 2013, **103**(6):e38-e46.
