# Supplementary material for: Passive sensing on mobile devices to improve mental health services with adolescent and young mothers in low-resource settings: the role of families in feasibility and acceptability
Source: BMC Med Inform Decis Mak. 2021 Apr 7;21:117. doi: 10.1186/s12911-021-01473-2 (PMC8025381; doi:10.1186/s12911-021-01473-2)
Supplement: Supplementary file 3 — Additional file 3. Consolidated criteria for reporting qualitative studies (COREQ). [file 12911_2021_1473_MOESM3_ESM.docx]

**Supplemental File 3. Consolidated criteria for reporting qualitative studies (COREQ): 32-item checklist**

Developed from:

Tong A, Sainsbury P, Craig J. Consolidated criteria for reporting qualitative research (COREQ): a 32-item checklist for interviews and focus groups. *International Journal for Quality in Health Care*. 2007. Volume 19, Number 6: pp. 349 – 357

| **No.  Item** | **Guide questions/description** |  |
| --- | --- | --- |
| **Domain 1: Research team and reﬂexivity** |  |  |
| *Personal Characteristics* |  |  |
| 1. Inter viewer/facilitator | Which author/s conducted the interview or focus group? | N/A |
| 2. Credentials | What were the researcher’s credentials? E.g. PhD, MD | MA, MPH, MSc, MD, PhD, |
| 3. Occupation | What was their occupation at the time of the study? | Researchers, Associate Professor, Assistant Professors, Interns |
| 4. Gender | Was the researcher male or female? | Both |
| 5. Experience and training | What experience or training did the researcher have? | BK, AL, AH have PhD degree and have supervised several PhD and Masters level students in conducting both quantitative and qualitative research. SM has MA and he was project coordinator. AP is a research associate with MSc in public health. AT, CI were interns. PB has MS and is IT professional.  RP is an Assistant Professor in psychiatric nursing. |
| *Relationship with participants* |  |  |
| 6. Relationship established | Was a relationship established prior to study commencement? | None of the participants had an established relationship with any of the authors prior to study commencement. |
| 7. Participant knowledge of the interviewer | What did the participants know about the researcher? e.g. personal goals, reasons for doing the research | Participants were informed that the researchers were interested in testing the sensing technology to aid the psychosocial intervention. |
| 8. Interviewer characteristics | What characteristics were reported about the inter viewer/facilitator? e.g. Bias, assumptions, reasons and interests in the research topic | A brief introduction about the study, organization and interviewer’s names and positions provided to the participants before starting the interview. Interviewer-related biases were not identified. |
| **Domain 2: study design** |  |  |
| *Theoretical framework* |  |  |
| 9. Methodological orientation and Theory | What methodological orientation was stated to underpin the study? e.g. grounded theory, discourse analysis, ethnography, phenomenology, content analysis | Thematic analysis |
| *Participant selection* |  |  |
| 10. Sampling | How were participants selected? e.g. purposive, convenience, consecutive, snowball | Convenience sampling was used. |
| 11. Method of approach | How were participants approached? e.g. face-to-face, telephone, mail, email | Face-to-face interviews were conducted. |
| 12. Sample size | How many participants were in the study? | 38 |
| 13. Non-participation | How many people refused to participate or dropped out? Reasons? | Two participants dropped out of the study due to other priorities and pressure from a husband. |
| *Setting* |  |  |
| 14. Setting of data collection | Where was the data collected? e.g. home, clinic, workplace | All interviews were conducted at home with initial screening at the health post. |
| 15. Presence of non-participants | Was anyone else present besides the participants and researchers? | No |
| 16. Description of sample | What are the important characteristics of the sample? e.g. demographic data, date | Adolescent and young women (15-25 years) showing symptoms of postnatal depression. |
| *Data collection* |  |  |
| 17. Interview guide | Were questions, prompts, guides provided by the authors? Was it pilot tested? | Interview guide was prepared and it was rigorously discussed among the authors. Probes were used to facilitate discussions. |
| 18. Repeat interviews | Were repeat inter views carried out? If yes, how many? | No |
| 19. Audio/visual recording | Did the research use audio or visual recording to collect the data? | Interviews were audio recorded. |
| 20. Field notes | Were ﬁeld notes made during and/or after the interview or focus group? | Field notes after the interview were also transcribed and translated and included in the dataset for the analysis. |
| 21. Duration | What was the duration of the inter views or focus group? | Interviews with women lasted for about 20-45 minutes. |
| 22. Data saturation | Was data saturation discussed? | No. |
| 23. Transcripts returned | Were transcripts returned to participants for comment and/or correction? | No |
| **Domain 3: analysis and ﬁndings** |  |  |
| *Data analysis* |  |  |
| 24. Number of data coders | How many data coders coded the data? | Four authors (SM, AP, AT and CI) coded the data. |
| 25. Description of the coding tree | Did authors provide a description of the coding tree? | Yes, the authors generated a coding system and codebook in MS Excel with domains, codes, and themes generated. |
| 26. Derivation of themes | Were themes identiﬁed in advance or derived from the data? | Themes were derived from the data. |
| 27. Software | What software, if applicable, was used to manage the data? | NVivo 12.0 |
| 28. Participant checking | Did participants provide feedback on the ﬁndings? | No |
| *Reporting* |  |  |
| 29. Quotations presented | Were participant quotations presented to illustrate the themes/ﬁndings? Was each quotation identiﬁed? e.g. participant number | Yes, quotations were presented to illustrate the themes/findings, and each quotation was identified with an anonymous participant code. |
| 30. Data and ﬁndings consistent | Was there consistency between the data presented and the ﬁndings? | Yes, there was consistency between the data presented and the findings. |
| 31. Clarity of major themes | Were major themes clearly presented in the ﬁndings? | Yes, major themes were clearly presented in the Results section using specific sections regarding each theme. |
| 32. Clarity of minor themes | Is there a description of diverse cases or discussion of minor themes? | Yes, minor themes were clearly presented in the Results section using specific sections regarding each theme. |
